# The Demographic and Socioeconomic Factors Predictive for Populations at High-Risk for La Crosse Virus Infection in West Virginia

**DOI:** 10.1371/journal.pone.0025739

**Published:** 2011-09-28

**Authors:** Andrew D. Haddow, Danae Bixler, Amy J. Schuh

**Affiliations:** 1 Department of Entomology and Plant Pathology, The University of Tennessee, Knoxville, Tennessee, United States of America; 2 Department of Pathology and Center for Biodefense and Emerging Infectious Diseases, The University of Texas Medical Branch, Galveston, Texas, United States of America; 3 West Virginia Bureau for Public Health, Charleston, West Virginia, United States of America; Universidade Federal de Minas Gerais, Brazil

## Abstract

Although a large body of literature exists for the environmental risk factors for La Crosse virus (LACV) transmission, the demographic and socioeconomic risk factors for developing LACV infection have not been investigated. Therefore, this study investigated the demographic and socioeconomic risk factors for LACV infection in West Virginia from 2003 to 2007, using two forward stepwise discriminant analyses. The discriminant analyses were used to evaluate a number of demographic and socioeconomic factors for their ability to predict: 1) those census tracts with at least one reported case of LACV infection versus those census tracts with no reported cases of LACV infection and 2) to evaluate significantly high-risk clusters for LACV infection versus significantly low-risk clusters for LACV infection. In the first model, a high school education diploma or a general education diploma or less and a lower housing density

were found to be predictive of those census tracts with at least one case of LACV infection. A high school or a general education diploma or less, lower housing density, and housing built in 1969 and earlier were all found to be predictive of those census tracts displaying high-risk clusters versus census tracts displaying low-risk clusters in the second model. The cluster discriminant analysis was found to be more predictive than the census tract discriminant analysis as indicated by the Eigenvalues, canonical correlation, and grouping accuracy. The results of this study indicate that socioeconomically disadvantaged populations are at the highest risk for LACV infection and should be a focus of LACV infection prevention efforts.

## Introduction

La Crosse virus (LACV) is a member of the California serogroup of viruses, genus *Orthobunyavirus*, family *Bunyaviridae*, and is the causative agent of LACV infections. Since its isolation in 1964 [1], LACV has become one of the most common causes of pediatric arboviral encephalitis in the United States [2], [3]. Although LACV has traditionally been associated with forested areas in the upper-Midwestern United States [4], the virus is the cause of an emerging disease in the Appalachian region of the United States [5], [6], [7], [8], [9], [10], [11].

The virus is maintained in nature through both vertical and horizontal transmission cycles involving vector mosquitoes and amplifying sciurid hosts [12], [13], [14]. The primary vector, the eastern tree-hole mosquito, *Aedes triseriatus* [15], [16] and the primary amplification hosts: the eastern chipmunk, *Tamias striatus*, the gray squirrel, *Sciurus carolinensis,* and the fox squirrel *Sciurus niger* [17], [18] maintain this cycle in focal areas where both vectors and amplification hosts are present. These areas of focal transmission are typically found within forested/vegetated areas or within peridomestic environments [19], [20], [21].

Since its recognition as a cause of human illness in 1964, LACV infections have been reported in 24 states [22], with the number of reported cases increasing in recent years in the Appalachian region of the United States [5], [6], [7], [9], [10], [11]. Following its detection in Appalachia, focal outbreaks of LACV infection have become a seasonally recognized cause of viral encephalitis in this region [5], [8], [11], [21], [23]. The majority of LACV infections are asymptomatic or present as a mild febrile illness, though a subset of the population develop severe disease, and present as either LACV meningitis, encephalitis, or meningioencephalitis [7], [9], [21], [24], [25], [26], [27]. These severe infections can result in a variety of short and long-term sequealae including seizures, behavioral changes, learning disabilities, and cognitive deficits [7], [25], [27], [28].

West Virginia has seen a large increase in the number of reported cases since 1987 [9] , and from 2000 to 2009 the state reported the largest cumulative number of cases in the United States (CDC unpublished). Although the environmental risk factors for transmission of LACV are well documented within the literature [6], [11], [19], [20], [21], [23], [29], [30], [31], [32], little is known about the demographic and socioeconomic risk factors for infection. Therefore, we performed two forward stepwise discriminant analyses to evaluate a set of demographic and socioeconomic factors for their ability to predict those census tracts in West Virginia with at least one reported case of LACV infection from 2003 to 2007 versus those census tracts with no reported cases of LACV infection, as well for their ability to predict high-risk clusters for LACV infection in West Virginia from 2003 to 2007 versus low-risk clusters for LACV infection.

## Methods

### Case data

La Crosse virus infection case data were investigated and collected by the West Virginia Department of Health and Human Resources from 2003 to 2007 because La Crosse virus infection (California serogroup viral disease) is a nationally notifiable infectious disease under the category: Neuroinvasive and Non-Neuroinvasive Domestic Arboviral Diseases [33], [34]. As such, these data are maintained and analyzed by the West Virginia Department of Health and Human Resources under this regulation. All personal identifiers were deleted to protect patient confidentiality before database construction and data release to the University of Tennessee for anonymous data analyses. Prior to the release of data, cases were classified as either confirmed or probable based on clinical and laboratory findings [34] by the West Virginia Department of Health and Human Resources. Confirmed and probable cases were combined for all analyses [8], [9]. There were 81 cases for which the patient's residence was available ranging in age from 0.42 years to 54.00 years (median  = 8.00 years; 1 case missing age). This research was deemed exempt from review and certification by the University's Institutional Review Board following review by the Departmental Review Committee under the University of Tennessee's guidelines for research involving human subjects.

### Demographic and socioeconomic variables

Demographic and socioeconomic variables were created from data obtained from the 2000 United States decennial census [35], and included population density, housing density, income, age, sex, education, and housing age (Table 1).

**Table 1:** Variables considered in the census tract and cluster discriminant analyses.

Variable	Description	Mean*	Mean†
Population density	Population per square kilometer	383.21	628.98
Housing density	Housing units per square kilometer	194.18	317.53
Income	Median household income	29,381.17	29,121.34
Race	Percentage of the population that is white	94.36	94.20
Age	Median age of the population	39.24	39.01
Sex	Percentage of the population that is male	48.40	48.10
Education	Percentage of the population with < HSD^1^/GED^2^	25.10	22.37
	Percentage of the population with HSD^1^/GED^2^	39.05	36.95
	3Percentage of the population with > HSD^1^/GED^2^	35.85	40.68
Housing	Percentage of housing built in 1969 and earlier	54.80	52.58
	Percentage of housing built 1970–1979	17.84	16.28
	Percentage of housing built between 1980–1989	13.26	10.26
	Percentage of housing built between 1990–2000	14.10	11.17

*Mean of the variables considered in the census tract analysis comparing those census tracts with one or more reported cases of La Crosse virus infection to those census tracts without any reported cases of La Crosse virus infection.

† Mean of the variables considered in the cluster discriminant analysis comparing those census tracts of significantly high-risk to those of significantly low-risk for La Crosse virus infection.

1 HSD: High school diploma.

2 GED: General education diploma.

3 >HSD/GED: Includes some college, associate degree, bachelor degree, graduate degree or professional degree.

### Geographic analysis

The census tract level was used for all analyses because it is more appropriate than larger geographic levels (e.g. county) for conducting spatial analyses of focal diseases [8], [36]. The cumulative incidence of LACV infection cases (number of LACV infection cases per 100,000 persons) were calculated for all census tracts in West Virginia (n = 466) using PASW 18 [37] for use in the spatial analysis. Evidence of spatial clustering was assessed using the global Moran's I statistic [38], and the Local Indicators of Spatial Autocorrelation (LISA) [39] using inverse distance spatial weights in GeoDa 0.95i [40]. Statistical significance for both the global Moran's I and the LISA statistics were tested using 9999 permutations. LISA statistic values were expressed as high-high (high-risk) or low-low (low-risk), indicating significant positive spatial autocorrelation. Geographic boundary files were downloaded from the United States Census, TIGER, Geodatabase [41], and cartographic displays were made using ArcView 9.3 [42].

### Discriminant analyses

Two forward stepwise Wilks' lamda discriminant analyses were performed to determine the demographic and socioeconomic factors that contributed the most to the discrimination between two groups. The first discriminant analysis was used to predict group membership in census tracts with no LACV infection cases versus census tracts with at least one case of LACV infection. The second discriminant analysis was used to predict group membership in high-risk clusters of LACV infection versus low-risk clusters of LACV infection. Table 1 provides a description and a mean value for each of the demographic and socioeconomic independent variables used in the census tract and cluster discriminant analyses, respectively. At each step of both the census tract and cluster discriminant analyses all independent variables were evaluated to determine which variable contributed the most to the discrimination between groups. Both stepwise procedures were guided by an F of 3.84 for inclusion and an F of 2.71 for exclusion. The F statistic has a numerator (df1) and a denominator (df2) degrees of freedom, which are used to obtain a significance level (p-value).

## Results

### Spatial analyses

**Figure 1:**
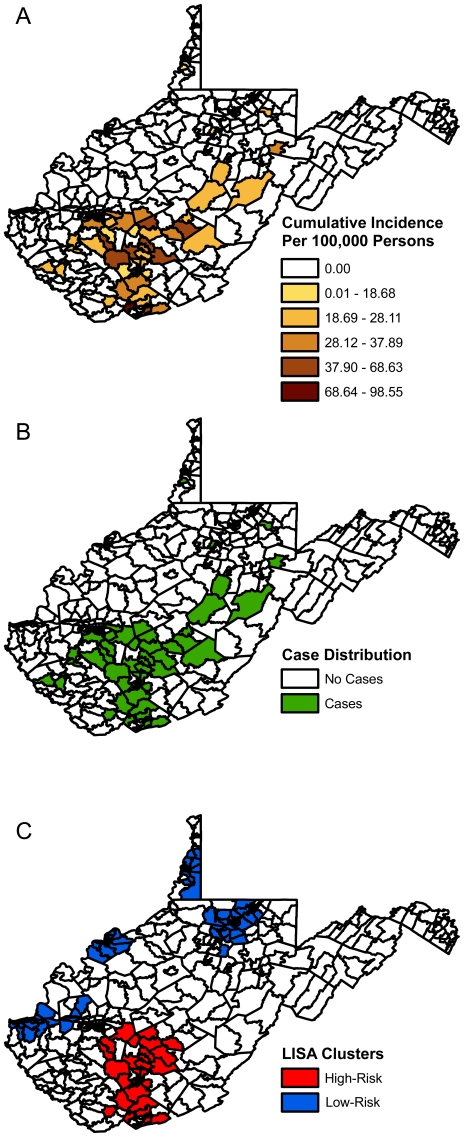
Distribution of the cumulative incidence, cases, and clusters of reported La Crosse virus infections in West Virginia census tracts from 2003 to 2007. (A) Cumulative incidence of La Crosse virus infections, (B) Distribution of cases of La Crosse virus infection, and (C) Significantly high- and low-risk clusters for La Crosse virus infection.

The cumulative incidence of LACV infection cases per 100,000 persons in those census tracts reporting cases ranged from 8.98 to 98.55 (median 29.42). Those census tracts reporting cases of LACV infections were located primarily within the south-central region of the state (Figure 1A). Counties with census tracts displaying cases were: Boone (n = 3), Clay (n = 10), Fayette (n = 10), Greenbrier (n = 1), Harrison (n = 1), Kanawha (n = 8), Logan (n = 1), McDowell (n = 1), Marshall (n = 1), Mercer (n = 9), Mingo (n = 1), Nicholas (n = 4), Preston (n = 1), Putnam (n = 1), Raleigh (n = 10), Tucker (n = 1), Upshur (n = 1), Webster (n = 1), Wyoming (n = 1).

The global Moran's I statistic detected evidence of significant spatial clustering at the census tract level with a computed global Moran's I value of 0.3375 (p = 0.0001). Furthermore, significant positive (high-high and low-low) spatial autocorrelation was observed at the census tract level using the LISA statistic. The cumulative incidence of LACV infection cases per 100,000 persons in those census tracts in the significantly high risk clusters (p<0.05) ranged from 8.98 to 98.55 (median 32.63) and in the significantly low risk clusters (p<0.05) was 0.00 (median 0.00). The high-risk clusters were observed in the south-central region of the state, while the significant low-risk clusters were observed in several regions typically corresponding to urban areas (Figure 1B). High-risk spatial clustering was observed in census tracts in the counties of: Boone (n = 1), Fayette (n = 10), Kanawha (n = 2), McDowell (n = 1), Mercer (n = 9), Nicholas (n = 2), Raleigh (n = 10), and Wyoming (n = 1), while low risk spatial clustering was observed in census tracts within in the counties of: Brooke (n = 2), Cabell (n = 29), Harrison (n = 10), Marrion (n = 18), Marshall (n = 7), Monogalia (n = 15), Ohio (n = 20), Putnam (n = 5), Taylor (n = 2), Wayne (n = 7), and Wood (n = 27).

### Census tract analysis

The first discriminant analysis compared those census tracts with no LACV infection cases to census tracts with at least one case of LACV infection. West Virginia is comprised of 466 census tracts, all of which were included in the analysis; 408 census tracts reported no LACV infection cases, while 58 census tracts reported at least one case of LACV infection. Table 2 shows the independent variables that remained in the final forward stepwise Wilks' lamda discrminant analysis model, the mean value for each independent variable according to the dichotomous dependent variable (census tracts with no LACV infection cases versus census tracts with at least one LACV infection case) and their standardized canonical discriminant coefficients, as well as selected model parameters including the Eigenvalue, the canonical correlation and the grouping accuracy.

**Table 2:** Independent variables retained following the discriminant analysis comparing census tracts with no reported cases of LACV infection to those census tracts with at least one reported case of LACV infection and selected model parameters.

Independent variable	Mean for census tracts		Standardized canonical discriminant function coefficients	Eigenvalue	Canonical correlation	Grouping accuracy (%)
	0 LACV cases	> 0 LACV cases				
< HSD^1^/GED^2^ (% of the population)	24.45	29.70	0.766	0.043	0.203	62.7
Housing density (units/km^2^)	211.46	72.59	−0.492			

1 HSD: High school diploma.

2 GED: General education diploma.

Based on the analysis, the percentage of the census tracts with less than a high school education diploma or general education diploma (Wilks' lambda: 15.421, df1 = 1, df2 = 464, p = 0.000) is the single best predictor for discriminanting between census tracts with no LACV infection cases and census tracts with at least one LACV infection case, followed by housing density (Wilks' lambda: 9.985, df1 = 2, df2 = 463, p = 0.000). A census tract is more likely to include at least one LACV infection case when it has a higher percentage of persons with less than a high school education diploma or general education diploma (29.70% of the population versus 24.45% of the population) and a lower housing density (72.59 housing units/km^2^ versus 211.46 housing units/km^2^) and The Wilks' lambda test (0.959) indicated that the Eigenvalue (0.043) was statistically significant (p = 0.000), suggesting that the model is a good fit for the data. The canonical correlation of 0.203 indicates a weak-positive relationship between the dependent variable (census tracts with no LACV infection cases versus census tracts with at least one LACV infection case) and the independent variables retained in the model (percentage of the population with less than a high school education diploma or general education diploma and housing density); squaring this value provides the percentage of variance explained in the dependent variable (4.12%). The standardized canonical discriminant function coefficients indicate the relative importance of the independent variables (percentage of the population with less than a high school education and housing density) in predicting census tracts with no LACV infection cases and census tracts with at least one case of LACV infection. These coefficients were used to write an equation for the discriminant function (discriminant function = −0.492 * housing density [housing units/km^2^] + 0.766 * percentage of the population with less than a high school education diploma or general education diploma) which can be used to predict a person's inclusion in a census tract with no LACV infection cases versus a census tract with at least one case of LACV infection. The group centroids (census tracts with no LACV infection cases  = −0.078; census tracts with at least one case of LACV infection  = 0.550) can be used to calculate a cut score (0.236) halfway between the two centroids. If a person's score on the discriminant function is above 0.236 then they most likely belong to a census tract with at least one case of LACV infection, whereas if their score is below 0.236 then they most likely belong to a census tract with no LACV infection cases. Overall, 62.7% of the sample was correctly classified into either census tracts with no cases of LACV infection versus census tract with at least one case of LACV infection, exceeding the value for classification based on chance (50%). At the individual group level, 61.0% of the population belonging to census tracts with no cases of LACV infection was correctly classified and 74.1% of the population belonging to census tracts with at least one case of LACV infection was correctly classified.

### Analysis of high and low risk clusters

The second discriminant analysis compared low-risk clusters for LACV infection to high-risk clusters for LACV infection. Within the state of West Virginia, 142 low-risk census tracts and 36 high-risk census tracts for LACV infection were identified. Demographic and socioeconomic independent variables used in this analysis include age, race, sex, population density, housing density, income, housing age and education. Table 3 shows the independent variables that remained in the final forward stepwise Wilks' lamda discriminant analysis model, the mean value for each independent variable according to the dichotomous dependent variable (low-risk clusters for LACV infection and high-risk clusters for LACV infection) and their standardized canonical discriminant coefficients, as well as selected model parameters including the Eigenvalue, the canonical correlation, and the grouping accuracy. Based on the analysis, the percentage of the population within the cluster with less than a high school education (Wilks' lambda: 44.164, df1 = 1, df2 = 176, p = 0.000) is the single best predictor for discriminanting between low-risk clusters for LACV infection and high-risk clusters for LACV infection, followed by the housing density of the cluster (Wilks' lambda: 32.056, df1 = 2, df2 = 175, p = 0.000), the percentage of housing within the cluster that was built in 1969 and earlier (Wilks' lambda: 24.469, df1 = 3, df2 = 174, p = 0.000) and finally the percentage of the cluster with a high school education diploma or general education diploma (Wilks' lambda 19.778, df1 = 4, df2 = 173, p = 0.000). A cluster was found to be at higher risk for LACV infection when it had a higher percentage of the population with less than a high school education diploma/general education diploma (30.70% of the population versus 20.25% of the population), a lower housing density (78.63 housing units/km^2^ versus 378.10 housing units/km^2^), a higher percentage of housing built in 1969 and earlier (54.46% versus 52.10%) and a higher percentage of the population with a high school education diploma or general education diploma (39.29% of the population versus 36.35% of the population). The Wilks' lambda test (0.686) indicated that the Eigenvalue (0.457) was significant (p = 0.000), suggesting that the model was a good fit for the data. Of note, this model of low-risk clusters versus high-risk clusters of LACV infection is more discriminating than the first discrminant model (census tracts with no cases of LACV infection versus census tracts with at least one case of LACV infection), as the Eigenvalue increased more than ten-fold. The canonical correlation of 0.560 indicates a strong-positive relationship between the dependent variable (low-risk clusters for LACV infection versus high-risk clusters for LACV infection) and the independent variables retained in the model (less than a high school education diploma, housing density, percentage of housing built in 1969 and earlier and percentage of the population with a high school education diploma or general education diploma); squaring this value provides the percentage of variance explained in the dependent variable (31.36%).

**Table 3:** Independent variables retained following the discriminant analysis comparing low-risk clusters for LACV infection to high-risk clusters for LACV infection and selected model parameters.

Independent variable	Mean for census tracts		Standardized canonical discriminant function coefficients	Eigenvalue	Canonical correlation	Grouping accuracy (%)
	Low-risk clusters for LACV infection	High-risk clusters for LACV infection				
< HSD^1^/GED^2^ (% of the population)	20.25	30.70	0.996	0.457	0.560	83.7
Housing density (units/km^2^)	378.10	78.63	−0.621			
Housing built in 1969 and earlier (% of housing)	52.10	54.46	0.312			
HSD^1^/GED^2^ (% of population)	36.35	39.29	−0.343			

1 HSD: High school diploma.

2 GED: General education diploma.

The correlation between the discriminant functions and the dependent variable is considerably stronger in this model (low-risk clusters of LACV infection versus high-risk clusters of LACV infection) compared to the first model (census tracts with no cases of LACV infection versus census tracts with at least one case of LACV infection). The standardized canonical discriminant function coefficients indicate the relative importance of the independent variables (percentage of the population with less than a high school education, housing density, percentage of the housing built in 1969 and prior, and percentage of the population with a high school or general education diploma) in predicting low-risk clusters for LACV infection and high-risk clusters for LACV infection. These coefficients were used to write an equation for the discriminant function (discriminant function  =  0.966*percentage of the population with less than a high school education + −0.621*housing density [housing units/km^2^] + 0.312*percentage of housing built in 1969 and earlier + −0.343*percentage of population with a high school or general education diploma, which can be used to predict a person's inclusion in a low-risk cluster for LACV infection versus a high-rick cluster for LACV infection. The group centroids (low-risk cluster for LACV infection = −0.339; high-risk clusters for LACV infection = 1.335) can be used to calculate a cut score (0.498) halfway between the two centroids. If a person's score on the discriminant function was above 0.498 then they most likely belonged to a high-risk cluster for LACV infection, whereas if their score was below 0.498 then they most likely belonged to a low-risk cluster for LACV infection. Overall, 83.7% of the sample was correctly classified into either low-risk clusters for LACV infection or high-risk clusters for LACV infection, exceeding the value for classification based on chance (50%). At the individual group level, 85.2% of the population belonging to clusters at low-risk for LACV infection was correctly classified and 77.8% of the population belonging to those clusters at high-risk for LACV infection was correctly classified. Compared to the first model (census tracts with no cases of LACV infection versus census tracts with at least one case of LACV infection), this model of low-risk cluster for LACV infection versus high-risk clusters for LACV infection is superior in its ability to accurately classify groups.

## Discussion

Since its isolation over 45 years ago, LACV has been increasingly recognized as an important cause of pediatric encephalitis. Previous work has examined the biology of vector species and hosts, viral evolution and pathogenesis, as well as the environmental risk factors for LACV transmission. Although socioeconomic and demographic factors of patients infected with LACV have been described [43], [44], no studies had investigated the demographic and socioeconomic risk factors predictive for LACV infection. In this study we utilized two forward stepwise discriminant analyses to investigate several demographic and socioeconomic factors for their ability to predict those census tracts in West Virginia with at least one reported case of LACV from 2003 to 2007 versus those census tracts with no reported cases of LACV, as well as for their ability to predict high-risk clusters for LACV infection in West Virginia from 2003 to 2007 versus low-risk clusters for LACV infection.

The results of this study found that the cluster discriminant analysis was more predictive than the census tract discriminant analysis as indicated by the Eigenvalues (model better fit for the data), canonical correlation (strong relationship between dependent variable and the discriminant function) and grouping accuracy (the cluster discriminant analysis was more effective because a higher percentage of the estimates were correct compared to the census tract discriminant analysis). These results are similar to those obtained in a previous study that employed a discriminant analysis to investigate environmental and social determinants of human risk of West Nile virus (WNV) infection in Chicago, Illinois [45]. In that study the authors determined that the discriminant analysis model that compared census tracts inside clusters to those census tracts outside clusters was more discriminating than the discriminant analysis model comparing those census tracts without reported human cases of WNV infection to those census tracts that reported at least one case of WNV infection. Our cluster discriminant analysis revealed that clusters at high-risk for LACV infection possessed a higher percentage of the population with less than a high school education diploma/general education diploma, a lower housing density, a higher percentage of housing built in 1969 and earlier and a higher percentage of the population with a high school education diploma or general education diploma.

Both the census-tract and cluster discriminant analyses indicated that possessing less than a high school or general education diploma was a risk factor for LACV infection in West Virginia. The cluster discriminant analysis also found that possessing a high school diploma or general education diploma was a risk factor. A lower level of education has been shown to be a possible risk factor for several other arboviruses, including West Nile and Saint Louis encephalitis viruses [46]. The finding that possessing a high school diploma or general education diploma or less is a risk factor for LACV infection is troubling, as these populations may have less access to and understanding of education efforts to prevent virus transmission. Due to the lack of awareness of LACV and its serious consequences to human health these populations are therefore less likely to be taking preventative steps to reduce virus transmission risk, such as wearing mosquito repellant, limiting exposure to mosquito populations during peaks of activity, filling tree holes to reduce mosquito larval habitats, and reducing artificial containers and standing water around human habitations.

The cluster discriminant analysis revealed that older housing was a risk factor for LACV infection in West Virginia as was also observed for WNV infection risk in Chicago [47], although the older housing in the Chicago study was built on a flood plain which could be considered a risk factor in itself. The association between older housing (built in 1969 or earlier) and an increased risk for LACV infection in West Virginia could be due to a variety of factors including the overall maintenance of houses and properties. When compared to newer houses, older houses are more likely to be in need of repairs or upkeep, and are more likely to experience blocked gutters, and/or the presence of artificial containers under patios or the structures foundation, thus in turn providing increased mosquito larval habitat. Older homes/properties may also suffer from higher frequencies of vegetation encroachment, established vegetation, and/or experience unkempt yards when compared to newer homes/properties. Furthermore, the lack of insect screening or the presence of damaged screens would likely occur at higher frequencies in older homes when compared to newer homes increasing the risk of mosquito and human contact.

Finally, our cluster discriminant analysis indicated that lower housing density (analogous to rural areas) was a risk factor for LACV infection in West Virginia. The association between LACV and forested areas is an established risk factor for LACV infection [11], [13], [19], [20], [21], [29], [32], [48]. Forested areas or isolated tree stands provide habitat for both the mosquito vectors and the amplification hosts. Additionally, areas of dense vegetation potentially mask artificial containers. Such areas are common in the peridomestic and rural environments associated with cases of LACV infection in West Virginia and elsewhere [11], [13], [19], [21], [23]. These areas of lower housing density also experience an absence of waste management/garbage pickup. These services, which are routine in urban areas are typically absent in rural areas within the state, especially in lower income rural areas. It is common for people to discard their trash at the edge of their property, in ditches, or by burning (metal containers are not damaged by burning). Unfortunately, these methods of waste disposal can create larval habitats, and water collecting in these discarded containers is typically rich in organic matter derived from decaying leaves providing immature mosquitoes with an abundant source of food.

Our finding that a lower housing density was a risk factor for LACV in West Virginia confirms a previous investigation that reported that 84% of the La Crosse encephalitis case patients in their study resided in the mountains of North Carolina, which are typically rural areas [44]. Our finding that a lower housing density was a risk factor for LACV infection in West Virginia is in contrast to that of WNV infection for much of the United States, where higher housing densities have been shown to be a risk factor for infection [45], [47], [49], although in Iowa, DeGoote *et. al*., [50] linked rural areas (analogous to lower housing density in our analyses) to an increased risk for WNV infection. These differences in the epidemiology of LACV and WNV are principally due to differences in vector and host species; the primary transmission cycle of LACV involves *Aedes* spp. and amplification in sciurid hosts, whereas the primary transmission cycle of WNV involves *Culex* spp. and avian hosts. The likeliest potential geographic overlap of these two viruses would occur within suburban forested environments of the eastern United States.

Several recent studies have examined the relationship between socio-demographic and/or economic factors and arbovirus transmission in the United States. Of note, the authors of a recent study examining the demographic factors related to West Nile virus and Saint Louis encephalitis infection cases in Houston, Texas observed that the region in their study area that experienced the highest number of artificial containers and the most severe disease was also the region that contained the most socioeconomically disadvantaged population, although these findings were not statistically significant [46]. This population exhibited a lower level of education, earned a lower household income, and had a higher level of poverty compared to those populations comprising the remainder of the study area. Similarly, in Maryland, though not statistically significant, cases of WNV infection were correlated with low income areas [51].

There are some limitations to the methodology employed in our study. The spatial analysis in this study was performed using the total population, to allow for comparison with census data, as census data was not exclusively available for the pediatric population. We used the LISA statistic to detect spatial clusters at significantly high-risk and low-risk for LACV infection. One drawback that can occur when using the LISA statistic for cluster detection is the issue of multiple comparisons, which would increase type I errors. These errors were not adjusted for, as adjustments for type I errors would increase type II errors [52], [53], in turn reducing the ability to detect truly significant clusters [53].

We used two forward stepwise discriminant analyses to determine the demographic and socioeconomic factors that contributed the most to the discrimination between the two groups: 1) census tracts with no LACV infection cases versus those census tracts with at least one reported case of LACV infection and 2) high-risk clusters for LACV infection versus low-risk clusters for LACV infection. The use of stepwise methodologies has been criticized for three problems that are inherent in their use: 1) incorrect degrees of freedom, 2) sampling error capitalization and 3) the failure to select the best subset of variables of a given size. Despite these criticisms we feel confident in our final models as they both retained less than a high school diploma/general education diploma and the housing density variables.

This is the first study to investigate the demographic and socioeconomic factors predictive for developing LACV infection. Our findings of a high school diploma/GED or less education, a lower housing density, and housing built in 1969 or earlier were found to be risk factors for LACV infection and are indicative of rural socioeconomically disadvantaged populations. These populations should be a focus of education efforts to prevent LACV transmission within endemic foci.
